# Suit for Malpractice

**Published:** 1849-09

**Authors:** 


### ARTICLE V

### *Suit for Malpractice.*

[We have received from Dr. Spencer the details of a suit for malpractice. The entire communication occupying more space than we are able to give, we present our readers with the following abstract, embracing the matter at issue, and the main points of the testimony.]

An action was brought for damages by Henry Glasford against Saml. Rifenbarick and Randolph Brearly; and, as set out in the Declaration, for unskilfully and ignorantly failing to reduce a luxated hip for the plaintiff.

The defendants set up in bar of damages, that the said injury complained of, was not a luxation, but a fracture of the neck of the femur, within the capsular ligament.

John Glasford, son of Plaintiff, deposed that his father in alighting from his horse, had his foot retained in the stirrup, and that the horse ran violently with him for a distance of six or eight rods. The patient, at the time of the accident, was 61 years of age---was carried into the house and visited within an hour by Dr. Rifenbarick, who found him lying on his back, with his limbs drawn up---examined him during a space of twenty minutes---said the joint was not luxated, but badly sprained. Witness thinks plaintiff did not request defendant to return, nor did defendant say he would. Plaintiff suffered severely for four or five weeks---that during this time witness went after Dr. R. who came and gave plaintiff a dose of salts ---went after the Doctor because plaintiff was feverish and costive. The Doctor called no more for some time, and then called in company with Dr. Brearly. When plaintiff got up, the injured limb was found to be too short, and this was the first intimation they had of the fact.

Wm. Glasford says he was at his father's when Dr. R. came---states, as to the examination, about what former witness did; but thinks the examination might have lasted half an hour. No measurement was resorted to. The leg is now about two inches shorter than the other, with the toes turned out.

Dr. McFarland called---is a physician and surgeon of twelve years practice---a graduate of Jefferson Medical College. Says there are four luxations of the hip: this is the first---he critically examined the plaintiff, and felt satisfied that he was correct---said the first luxation was that in which the force applied below, drove the thigh bone upwards and backwards on the *dorsum ilii*---thinks this view of the case accounts for the turning of the toe out, as well as the shortening of the limb. In this luxation, the toe at first turns in, but by long continued use it turns out. Witness was asked if this might not be a fracture. Thought not, from the nature of the injury, and the frequency of luxations compared with fractures, and that upon the day before he had heard [no?] *crepitus* upon moving the limb. Witness was asked what the treatment should be if the injury were within the capsular ligament? Said the pullies ought to be applied.

Dr. Moore called, Is a graduate: has been three years in practice: testified as to the luxations of the thigh about what the former witness stated; and coincided with him in supposing this to be a luxation. Thinks that in this luxation the toe at first turns in, but afterwards out. Thought it possible that the injuiy might be a fracture without the capsular ligament, not within; but believed it to be the first luxation,

Dr. Spencer called. Is a physician and surgeon of about twelve years practice. Illustrated the four luxations upon the skeleton. Thinks this is neither of the four: knows of no luxation in which the injured limb is shortened two inches, with the toe turned outward. The anatomy of the parts precludes the possibility of such an appearance, unless the injuries were accompanied by great muscular lesion. Thinks this injury is a fracture of the neck of the femur, and of course within the capsular ligament. A competent surgeon can distinguish a fracture by the facility of extending the injured limb to an equal length with the other, by crepitation, and by the limb being again retracted when the extending force is withdrawn. The probabilities of a fracture to an old person are *much greater* than a luxation---did not believe it would be easy to pull a man's leg shorter by drawing at the foot, nor that violently jerking at the limb would produce the first luxation. From the symptoms it would of course be absurd to suppose that this was the second, third or fourth dislocations, consequently concludes it must be a fracture.---- Would not undertake to say whether or not the examination of the injury by Dr. Rifenbarick was or was not sufficient---any examination was a good one that would satisfy the surgeon as to the nature of the injury---the rule was to lay the patient on his back, and pass a tape down the median line.

Supposed an injury of this sort, in an old person, to be incurable, and that the toe always turned out. In charging the jury, the judge remarked that before they could find for the plaintiff, they must be clearly satisfied that the injury complained of was a luxation; as this was the only specification in the declaration, and if they could entertain any reasonable doubt as to its nature they must find for the defendant. The jury then retired, and after five or six hours returned with damages of $200 for the plaintiff.

[Our opinion has been asked in this case; we can give it in few words. We cannot imagine the possibility of a luxation of the head of the femur on the dorsum of the ilium, with the toe turned outwards. This was doubtless a fracture, whether within or without the capsular ligament is a matter of no consequence in the present case, neither is it necessary for us to state whether or not the treatment by the attending physician was proper or not. We have no hesitation in pronouncing the verdict in this case to be unrighteous and illegal; in which opinion we are fully sustained by the charge of his Honor the Judge.—EDS.]

